# Animal taxa contrast in their scale-dependent responses to land use change in rural Africa

**DOI:** 10.1371/journal.pone.0194336

**Published:** 2018-05-08

**Authors:** Stefan Hendrik Foord, Lourens Hendrik Swanepoel, Steven William Evans, Colin Stefan Schoeman, Barend Frederik N. Erasmus, M. Corrie Schoeman, Mark Keith, Alain Smith, Evans Vusani Mauda, Naudene Maree, Nkhumeleni Nembudani, Anna Sophia Dippenaar-Schoeman, Thinandavha Caswell Munyai, Peter John Taylor

**Affiliations:** 1 Department of Zoology and Centre for Invasion Biology, School of Mathematical & Natural Science, University of Venda, Thohoyandou, South Africa; 2 South African Research Chair on Biodiversity Value and Change and Centre for Invasion Biology, School of Mathematical & Natural Science, University of Venda, Thohoyandou, South Africa; 3 Global Change and Sustainability Research Chair (GCSRI), University of the Witwatersrand, Johannesburg, South Africa; 4 School of Life Sciences, College of Agriculture Engineering and Science, University of KwaZulu-Natal, Durban, South Africa; 5 Centre for Wildlife Management, University of Pretoria, Pretoria, South Africa; 6 School of Life Sciences, College of Agriculture Engineering and Science, University of KwaZulu-Natal, Pietermaritzburg, South Africa; 7 School of Life Sciences, College of Agriculture Engineering and Science, University of KwaZulu-Natal, Westville, South Africa; University of Waikato, NEW ZEALAND

## Abstract

Human-dominated landscapes comprise the bulk of the world’s terrestrial surface and Africa is predicted to experience the largest relative increase over the next century. A multi-scale approach is required to identify processes that maintain diversity in these landscapes. Here we identify scales at which animal diversity responds by partitioning regional diversity in a rural African agro-ecosystem between one temporal and four spatial scales. Human land use practices are the main driver of diversity in all seven animal assemblages considered, with medium sized mammals and birds most affected. Even the least affected taxa, bats and non-volant small mammals (rodents), responded with increased abundance in settlements and agricultural sites respectively. Regional turnover was important to invertebrate taxa and their response to human land use was intermediate between that of the vertebrate extremes. Local scale (< 300 m) heterogeneity was the next most important level for all taxa, highlighting the importance of fine scale processes for the maintenance of biodiversity. Identifying the triggers of these changes within the context of functional landscapes would provide the context for the long-term sustainability of these rapidly changing landscapes.

## Introduction

Anthropogenic changes such as agricultural intensification and urbanization often have negative impacts on biodiversity and its associated ecosystem function [1]. Large areas in the tropical savannas of southern Africa are rapidly being transformed into rural agro-ecosystems which is comprised of a mosaic of settlements, agricultural fields and rangelands. Local communities are still largely dependent on these natural resources including fuel wood which is being harvested at rates that may be unsustainable and that may impact biodiversity through changes in vegetation structure, e.g. through loss of large trees with hollow cavities suitable for roosting animals [2, 3].

Implementing conservation management within these land use developments might include “land-sparing”, which is often advocated over “land-sharing” (wildlife-friendly farming) based on the assumption that agroecosystems harbor negligible biodiversity or ecosystem services [4]. The land sharing versus land sparing debate has been informed mostly by studies in developed (largely temperate) countries and the situation in small-holder agroecosystems in developing (predominantly tropical) countries is less understood.

Managers and policymakers should focus on biodiversity as an umbrella concept. Not only because of its intrinsic value, but because more diverse systems improve function [5], enhance multifunctionality [6] at all trophic levels and habitats [7], and ensure ecosystems resilience at landscape scales [8]. However, conservation planning initiative are often done at coarse scales and neglect finer scale patterns which result in suboptimal solutions particularly in heterogeneous landscapes [9]. Beta diversity stands central to biodiversity, as it measures the spatial and temporal distribution of species, which can be used to determine the scales that are significant for generating this diversity, and ultimately it conservation [10]. Organisms and the processes they respond to change with scale [11, 12], and such scale dependence should therefore affect ecosystem function in a landscape. Factors affecting diversity also varies with scale [12]. Veech & Crist [13] has provided a partitioning framework to identify the scales that are important to the overall diversity (gamma diversity) in a region that would allow for scale-specific interventions that could either maintain or maximize diversity.

The theoretical underpinnings of partitioning allow for the test of two null hypotheses, which are: 1) whether there are significant intraspecific aggregation between samples at a specific scale; and 2) the spatial differentiation between samples at different scales [14]. Partitioning could be applied to a range of diversity metrics, e.g. richness and Shannon diversity as they measure different aspects of assemblage structure [14].

Our study aimed to assess the impacts of land transformation on biodiversity across a gradient of increasing human disturbance in two such areas of the Limpopo province, South Africa, where rapidly expanding small-holder agro-ecosystems encroach on unprotected “Critical Biodiversity Areas” identified by a recent provincial conservation plan [15]. This involved quantifying the scale dependent response of seven important vertebrate and invertebrate ecosystem providers in and around two rural settlements. Bats, birds, medium sized and small mammals (rodents and shrews) comprise the bulk of terrestrial vertebrate diversity in landscapes and their functional significance has been shown in several studies [16, 17]. Arthropods contrast with these vertebrate taxa in their smaller body size, thermal requirements [18] and the scale at which they function [19]. In the vast majority of terrestrial habitats, ants, spiders, and beetles dominate assemblages and provide essential ecosystem services [20].

Here we aim to develop our understanding of scale dependent diversity in these agro-ecosystems. Specifically, our study aims to assess the scale specific response of the diversity in these seven animal taxa. We did this by quantifying the proportional contribution of four spatial and one temporal hierarchical level to total diversity. We tested at which levels species are spatially aggregated and whether observed patterns of diversity are the result of non-random groups of species for each of these levels. We predicted that land use in the region plays a significant role in explaining the diversity of all taxa. We also predicted that the role of the temporal and spatial levels in generating diversity will vary between taxa.

## Materials and methods

### Study site

**Fig 1:**
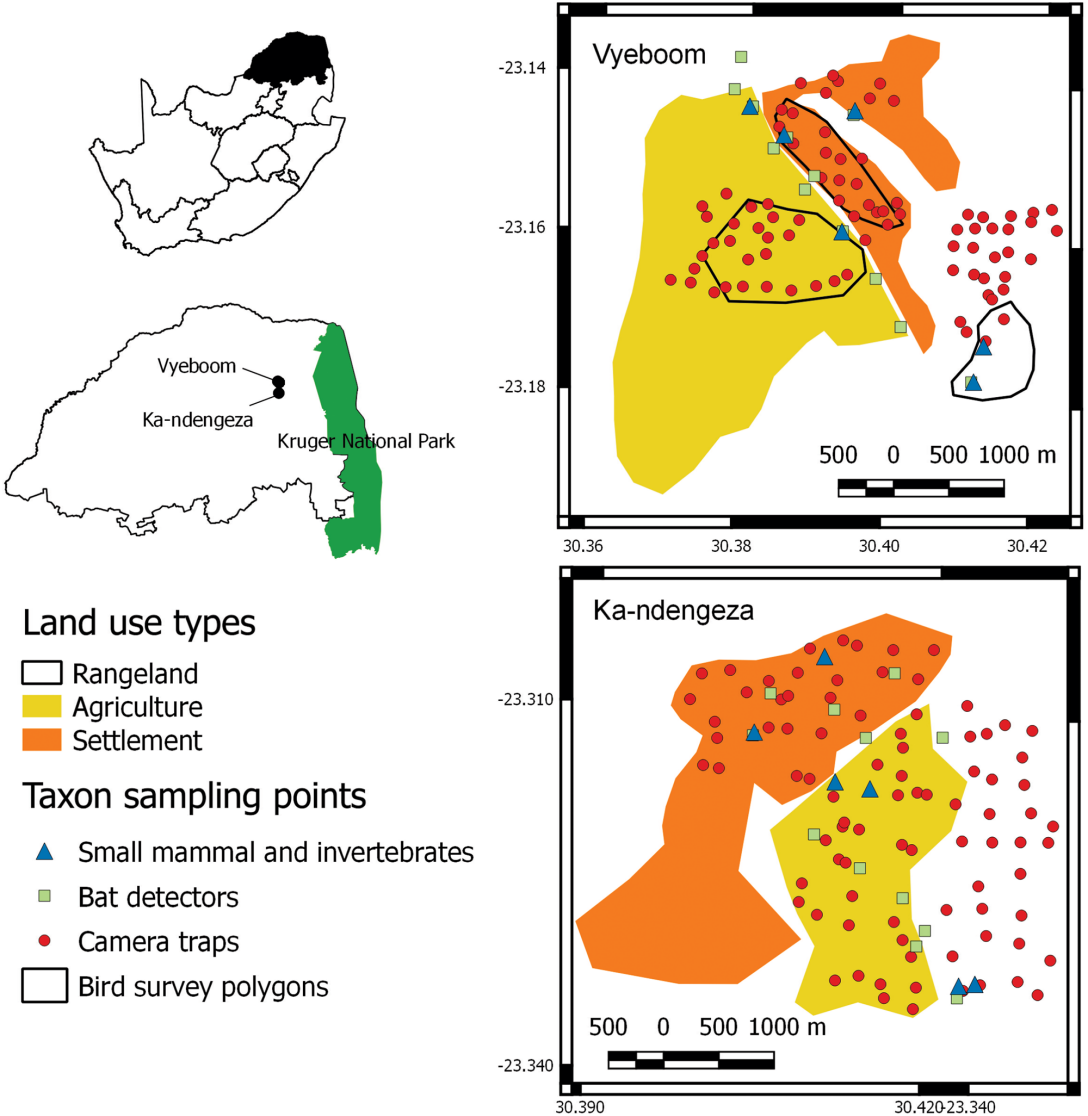
Map of study area indicating the three land use types and aerial photographs of each. Invertebrate and small mammal sampling grids are represented by their centroids, bird transects were conducted in Vyeboom and are represented by polygons. Point localities are given for camera traps and bat detectors.

This study was done in in the northeastern corner of South Africa among human settlements that border the Kruger National Park (Fig 1). QGIS 2.18.3 [21] was used to produce a study area map and political boundaries were taken from www.naturalearthdata [22]. The area is a remnant of the Apartheid-era zonation policies (former Bantustans) and is characterized by poverty, high human densities, food insecurity and environmental degradation [23]. The land use history of the study sites is well known. Historically the entire area was subjected to browsing and grazing by wild herbivores and occasional grazing from low-density Nguni cattle. Over the past forty years the area has been under traditional pastoralist management, but in the last 20 years rangeland for cattle at low densities have given way to subsistence cropland and emergent villages. Large herbivore stocking rates in rangeland sites fell within the limits predicted by the relationship between mean annual rainfall and large herbivore biomass in African savannas [24]. Such low-density-rangelands once characterised much of the continent’s savannas, as confirmed by a study that employed a combination of modelling and satellite photographic surveys across African savannas, showing that human densities of over 25 people per km-2 have been associated with shifts from low-density rangeland use to formation of cropland and villages [25].

We focused on areas in and around two villages, Vyeboom and Ka-Ndengeza (Fig 1). The first community, Vyeboom village (S 23.14407°, E 30.37657°), has a total population of around 5026 and an area of 6.1 km^2^ [26, 27]. The second community, Ka-Ndengeza village (S 23.30389°, E 30.40779°), has a population of around 3579 people and an area of 5.3 km^2^. Both villages can be divided into three main areas: croplands, communal grazing and human settlements (Fig 1). Semi-subsistence agriculture is mainly practiced, with crops including maize and peanuts, but vegetables like pumpkins, squashes and beans are also produced. A small proportion of community members also have macadamia and mango trees. Communal grazing areas or rangelands are also used for harvesting of a wide range of natural resources such as fuelwood, fruits and edible wild herbs. These resources are an important component of household resilience and are often more important than livestock grazing [28].

### Taxon sampling

Animals were collected under permit no. CPM- 005–00005, provided by Department of Environmental Affairs, South Africa. We sampled each taxon based on recognized sampling techniques, which included live trapping (Sherman traps—for rodents), camera traps (medium sized mammals), pitfall traps (invertebrates), acoustic sampling (bat detectors—bats) and time transects (birds). All surveys were conducted in the early dry and generally cold season of 2014 (mid-March to early May) and repeated the middle of the following wet and hot season of 2015 (February), except for camera trapping that was only done once during the dry and cold period (May to July 2014) when camera trapping is at its most efficient. Birds were sampled during both surveys but only in Vyeboom village.

#### Small mammal trapping (Rodents and Shrews)

We deployed standard rodent trapping protocols. In each land use we deployed a total of six replicates, two per village, for a total of 12 replicates (Fig 1). Replicates within a land use were at least 300 m apart. Each replicate consisted of 49 Sherman traps in a 7x7 grid and traps were 10 m apart. Each trap was baited with a rolled oats and peanut butter mix. Traps were deployed for a minimum of 5 days. Trapping was done in both the winter of 2014 (8 April to 8 June) and the summer of 2015 (3–12 February). Each trapped rodent was weighed, sexed, age and breeding condition estimated and then uniquely toe clipped [29]. We monitored captured rodents for up to 5 days in each survey (e.g. length of mark recapture study). Impact of trapping and handling were negligible on rodent survival (mean = 0.4% SE = 0.5% of marked rodent died/survey) and our results concur with others who found low impact of toe-clipping on rodent survival [29, 30]. All species caught are classified as least concern by IUCN and are therefore not protected [31]. Rodents were identified to species level using published keys [31].

#### Camera trapping

We followed an occupancy based framework for closed populations in our camera trapping setup [32]. To maintain spatial independence we first extracted small carnivore home range data from published sources [33] for small carnivores we expected to occur at the study site. Based on the median home range size we overlaid the study area with a 300m × 300m grid. For analytical purposes, each camera trap was considered to be a replicate and therefore independent. We deployed camera traps for nine days in each of the land use types (30 grids) and then moved camera traps to the next land use (29 May to 20 June at Ka-Ndengeza & 18 June to 8 July 2014 at Vyeboom; Fig 1). We used preliminary detection probabilities taken from pilot studies that informed sampling layout and time schedule based on Mackenzie and Royle [34]. In the settlement we deployed Black flash cameras (Cuddeback, Ambush 1194, Game Cameras) to minimize impact on inhabitants. In the agricultural and grazing area we deployed white flash cameras (Cuddeback, Ambush 1170, Game Camera). All camera traps affect detection among mammals [35], even infrared camera traps [36]. As such we believe that deployment of both back (infrared) and white flash camera traps will induce a similar bias among mammals detected. Camera traps were placed in well-defined animal paths or vehicle roads, 15cm above the ground and programmed to run for 24 h with a one minute delay between detections. Images we downloaded onto an image database (Camera Base [37]) for further analysis.

#### Bat detection

We used six Sony Meter (SM) BAT2+ (Wildlife Acoustics) bat detectors mounted on 4 m high flagpoles and set for between 3–5 nights (from dusk to dawn) at each passive recording station (12 per village for Vyeboom and Ka-Ndengeza, Fig 1). In total, 161 detector-nights were recorded. The detectors were set to record full-spectrum WAV files that were converted to Anabat zero-crossing (ZC) files using Kaleidoscope version 1.1.15 (www.wildlifeacoustics.com) and analysed in Analook version 4.1t. Recording stations were set as far apart from each other as possible and distributed as evenly as possible between habitats (settlements, agricultural fields and rangelands, Fig 1). Because of the limited existing road network required for access and the relatively small areas sampled around villages, some sites were situated <500 m apart. To reduce sample dependence and to classify the foraging habitat surrounding each station, we used Google Earth images to classify the proportion of the three main land uses in a circular buffer area of radius 1 km around each point. The dominant land use type (>60%) was assigned to each point. For three points at Vyeboom and two at Ndengeza, no land use types dominated >60%, and these points were excluded from further analyses to minimise error when testing for beta diversity due to dominant land use. We chose a radius of 1 km to accommodate the daily home range of most species of local bats. Based on radio-tracking data, clutter-feeding bats (e.g. *Nycteris thebaica*) typically move less than 1 km per night in commuting between roosts and foraging sites [38]. Although clutter-edge bats (e.g. *Scotophilus* spp.) may move between 1–3 km per night between roosts and feeding areas (e.g. in riparian zones), they tend to spend most of their time feeding in a smaller core area [39, 40]. Similarly, open-air feeders such as molossid bats (e.g. *Otomops martiennseni*) may move > 10 km per night to commute between roosts and feeding areas yet they also prefer smaller feeding areas [41]. To account for variation in bat activity due to multiple passes by the same individuals (e.g. around roosts), we followed Miller [42] in calculating an index based on the number of minutes recorded for each species per night.

Given the high richness of bats in the region, no automated bat identifier software is currently available to identify bats from their calls in the savanna biome of southern Africa, hence manual identification by an expert is the most reliable method [43]. The huge number of calls we collected over 161 detector-nights (>30,000 “species-minutes”) meant that manually identifying them all was impractical, thus we manually classified a subset (two nights each of eight sites per village for 2014 only) based on the reference call library developed by Taylor et al. [43] and used this sample of identified calls to investigate the reliability of using filters and scans in Analook W version 4.1t (Titley Electronics, www.hoarybat.com). Taylor et al. [43] identified pairs and groups of species from the savanna biome of northern South Africa which could not reliably be identified due to overlap in call parameters, and they advocated defining such as “species-groups” in acoustic surveys to minimise uncertainty. We followed a similar approach, involving even more aggregation of acoustically-similar species-groups, to come up with a final list of 13 species-groups which could be identified with minimal error using scans and filters in Analook. These 13 “acoustically-robust” groups comprised 21 distinct species identified manually by PJT (S1 Table). All of these species except *Otomops martiensseni* and *Chaerephon ansorgei* have been captured in the region by PJT (Taylor et al. [43]). Following Schoeman and Jacobs [44] and Taylor et al. [45], and because of the close relationship between families and subfamilies of bats and foraging groups, we classified each species-group into one of three foraging groups: clutter (Nycteridae, Rhinolophidae, Hipposideridae, Kerivoulinae), clutter-edge (Vespertilininae and Miniopteridae) and open-air feeders (Molossidae and Emballanuridae).

#### Bird surveys

Three timed transect counts (TTC), per habitat and per season (June 2014 and January 2015), were walked, each lasting three hours (Fig 1). Each timed transect was considered a replicate in our analysis. All birds seen and heard within a distance of 100 m either side of the transect route were included in the survey. The advantage of TTCs is that there is no restriction on the route walked, or length of the route, while time not distance was used to standardise effort.

#### Invertebrate sampling

Epigeal invertebrates were sampled using pitfall traps. Grids were set out at the exact same position as for small mammals. There were therefore two grids per land use per village, grids in a land use were at least 300 m apart to prevent pseudoreplication and each grid was therefore considered a replicate. Each grid represented a replicate which consisted of paired pitfall traps at 0 m, 1.5 m, 2 m and 2.5 m apart, with 15 m spacing between trap arrays (following Zhao *et al*. [46]). Pitfall traps were left open for five consecutive days. Our sampling design thus resulted in a total of 48 pitfalls × 3 land use types × 2 replicates = 576 pitfall per traps session. We trapped during dry season (June 2014) and again in the wet season (January 2015). We limited disturbance of the soil surface to the minimum and returned sites to their original state. Pitfall (ø 62 mm) traps contained 50% propylene glycol solution which does not attract nor repel ants [47]. Traps were removed after five days and ethanol was added as preservative. Ant samples were sorted and washed in the laboratory and preserved in 99.9% or 96% ethanol, and identified to morpho-species and where possible to species level by CSS (beetles), CTM (ants) and ASD (spiders) authors.

### Statistical analysis

Total observed richness and evenness (Shannon) were partitioned as:

$$\gamma_{obs}=\alpha +\beta_{point}+\beta_{replicate}+\beta_{land\;use}+\beta_{region}+\beta_{season}$$

where α is the mean alpha diversity per pitfall/camera trap/bat detector/Sherman trap/bird transect, β_point_ is the between-trap/transect beta diversity, β_replicate_ is the between-replicate beta diversity, β_landuse_ is the between land use beta diversity, β_region_ is the between village beta diversity and β_season_ is the beta diversity between dry and wet seasons. Birds were sampled in one village only, therefore total richness was partitioned into γ_obs_ = α + β_point_ + β_replicate_ + β_landuse_ + β_season_. Medium sized mammals (camera traps) were sampled once and partitioned into γ_obs_ = α + β_point_ + β_replicate_ + β_landuse_ + β_region_. Bat sampling contained no replicates and was partitioned into γ_obs_ = α + β_point_ + β_landuse_ + β_region_ + β_season_.

#### Aggregation and diversity hypotheses

We tested two hypotheses. First we tested for non-random intra-specific aggregation by comparing the observed richness contributed by a specific hierarchical level with that of a null model generated by randomly allocating individuals to the lowest hierarchical level [48]. If individual species are aggregated within different resource patches or habitats, within habitat diversity will be lower than expected and among-habitat diversity greater than expected. The analytical approach used here is referred to individual-based additive partitioning and was done using the function ‘adipart’ in vegan [49].

In the second hypothesis we tested whether similar diversity would be observed at a specific hierarchical level (n) if samples of the hierarchical level below (n-1) were randomly assigned to categories in level n, i.e. is the diversity observed at a specific scale merely the result of the sampling design [14]. The analytical approach here is referred to as additive sample-based partitioning and was done with the function ‘s. based’ [50]. Both these hypotheses were tested for both richness and evenness (Shannon diversity) measures of diversity and statistical modelling was done in R [51].

## Results

We detected 45 mammal (22 bat, 19 medium sized mammal [including domestic cat and dog], 12 rodent [small mammal]), 139 bird, 116 spider, 124 ant and 54 beetle species.

### Individual based partitioning of gamma diversity

**Fig 2:**
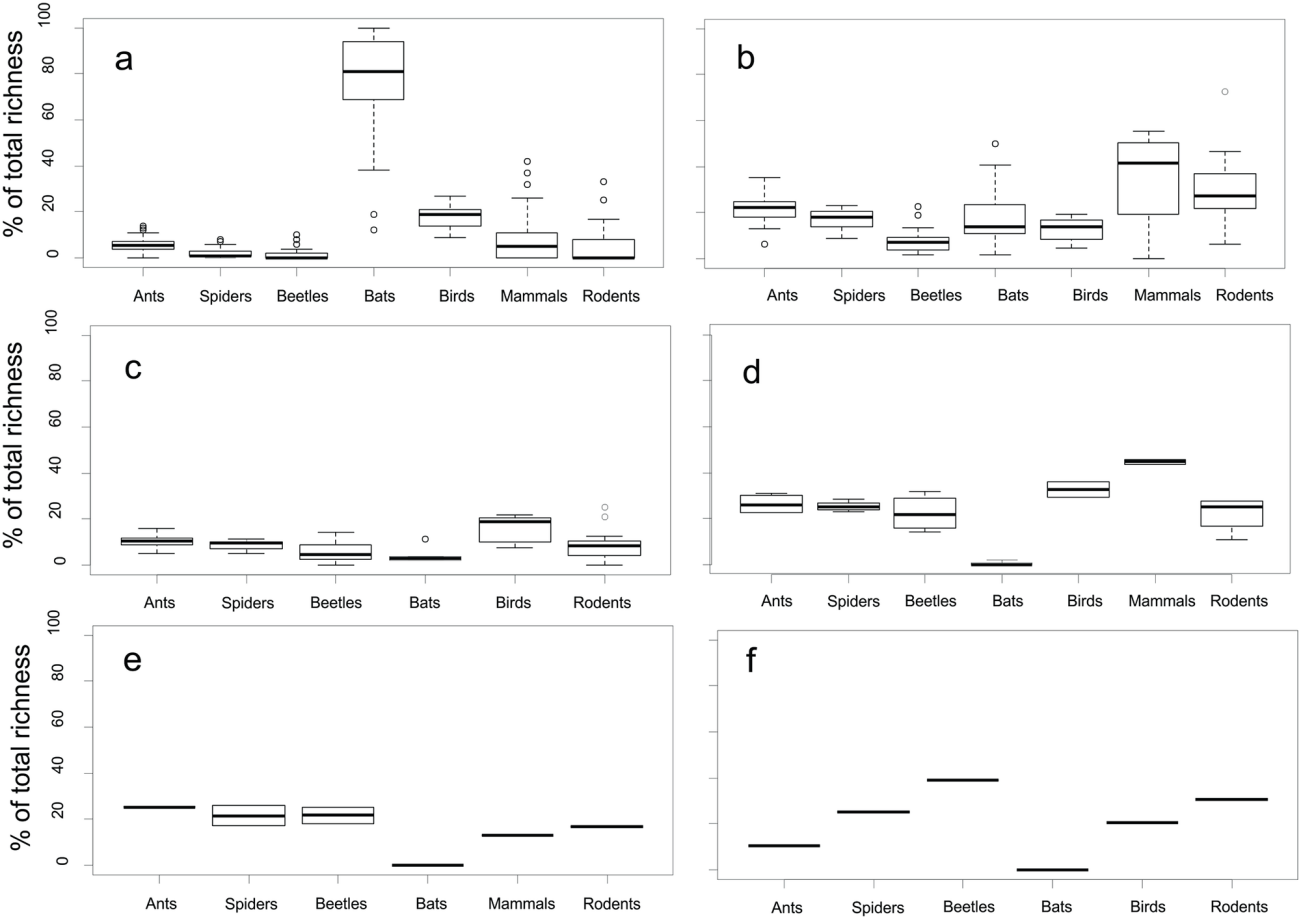
Relative contribution of a) α and b) β_point_, c) β_replicate_, d) β_landuse_, e) β_region_, f) β_season_ diversities to the γ-diversity of each taxon.

**Fig 3:**
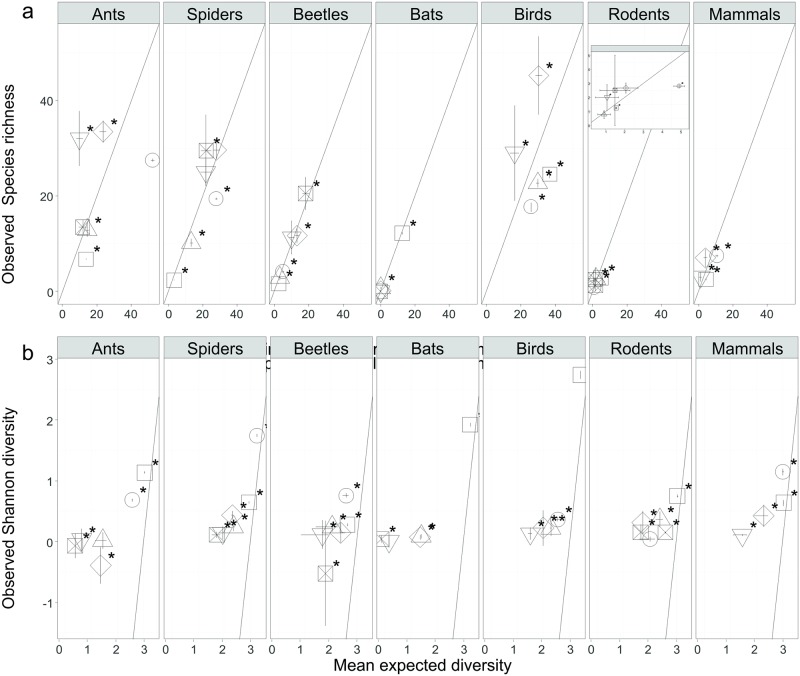
Results of individual-based partitioning of species richness (A) and Shannon diversity (B) plotted against means of null distributions for randomization tests. Results of hypothesis tests are listed in S1 Table. Statistically significant departures p < 0.05 from equal observed and expected components (45°_ line) are indicated by an asterisk. Vertical error bars are 2 SE of the mean of the observed sample distribution, and horizontal error bars are the critical upper and lower values that encompass 95% of the null distribution obtained from 999 randomizations. Inset: enlarged view of rodent partitioning. X-axis of estimated Shannon diversity is on a log scale.

Almost all of the regional bat richness was accounted for by richness at point localities (Figs 2 and 3a). Among point (within replicate) diversity was important for all taxa, particularly mammals and rodents (Figs 2 and 3a), whereas turnover between replicates was relatively low (< 10%) across all taxa, except birds (Figs 2 and 3a). Land use was very important in driving diversity, particularly for mammals and birds (> 30% of the total diversity) (Figs 2 and 3a). Regional diversity (turnover among villages) was more important for invertebrate taxa (Figs 2 and 3a). The effect of seasonality varied between individual taxa; beetles in particular, and birds and spiders to a lesser extent, had considerable seasonal turnover, while ants and bats had very little (Figs 2 and 3a).

The significantly lower than expected contribution to gamma diversity by samples taken at the finest scales of this study point to intraspecific aggregation of individual species at these levels (Fig 3a). Similarly, the contribution among higher scales were consistently higher than expected by chance (S2 Table). Small epigeal invertebrates had more than 60% of their turnover explained at larger scale (land use and larger). A smaller proportion (< 50%) of these processes accounted for the vertebrate diversity. Turnover among land uses made the second largest proportional contribution to gamma diversity for all taxa (Fig 3a), in close association with seasonality (beetles, spiders and birds), turnover among points (small and medium sized mammals) and villages (ants).

Point and among point evenness, made the largest contribution to overall evenness (Fig 3b). Point diversity’s contributions were not significant for any of the taxa, while the contribution of among point diversity varied between significantly larger for ants, beetles, bats and birds to significantly smaller for spiders and rodents (Fig 3b). All the other levels made significant, but relatively small, contributions to overall Shannon diversity, except for among land use (ants and beetles), and among replicates (rodents), which made negative contributions.

### Sample-based partitioning of gamma diversity

**Fig 4:**
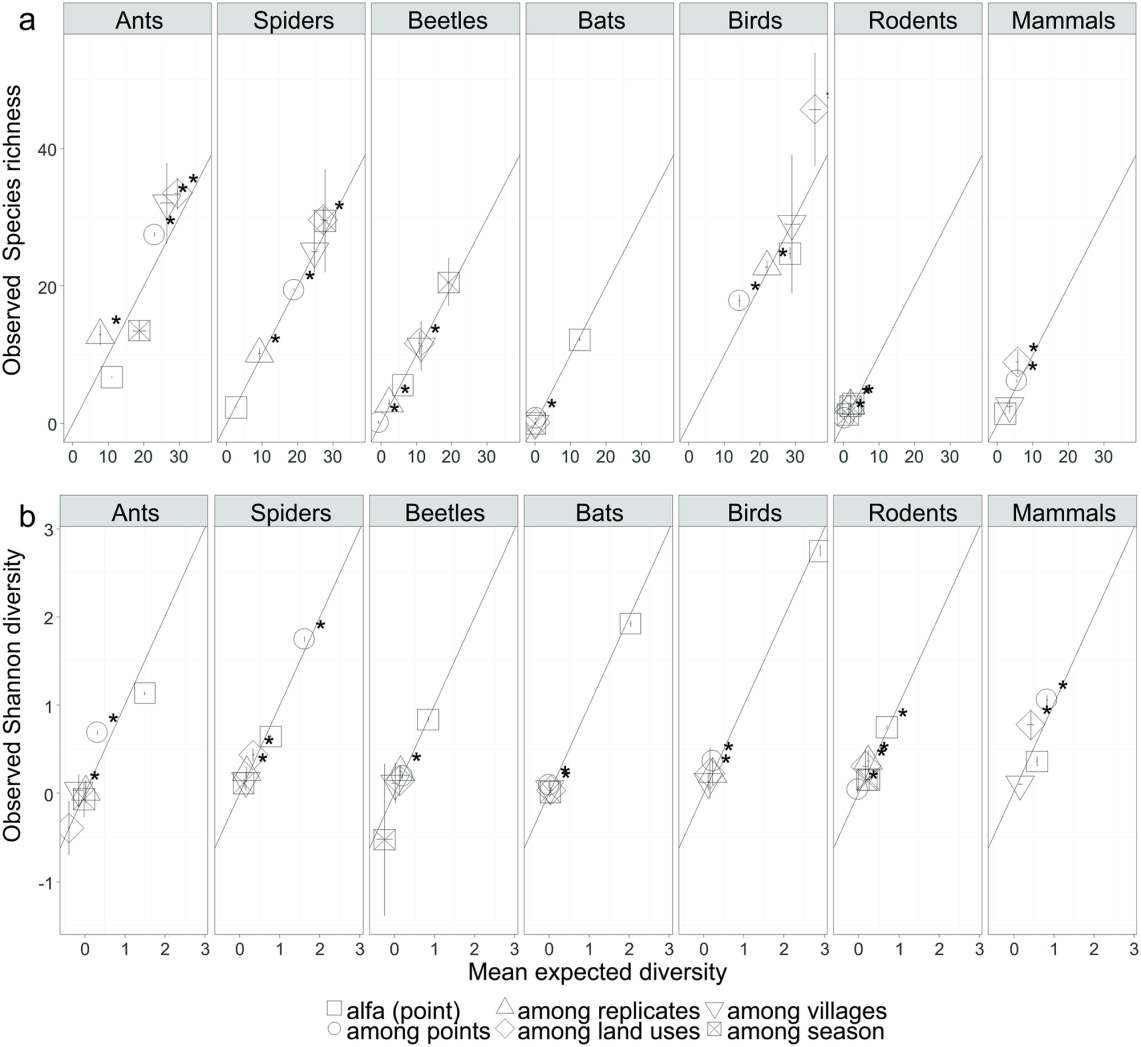
Observed richness (A) and Shannon diversity (B) for all taxa plotted against means of null distributions for sample-based randomization tests on species richness (A) and Shannon index (B) of diversity. Results of hypothesis tests are listed in S2 Table. Null distributions were obtained from 999 randomizations. Statistically significant departures p < 0.05 from equal observed and expected components (45°_ line) are indicated by an asterisk. Vertical error bars are 2 SE of the mean of the observed sample distribution, and horizontal error bars are the critical upper and lower values that encompass 95% of the null distribution obtained from 999 randomizations.

Land use was the only scale that consistently made a significantly larger than expected contribution to gamma diversity (Fig 4a and S2 Table). Observed richness was significantly positive for all the higher levels. The largest proportional contribution to total richness was made by larger scales (land use and village) in ant, spider, beetle, bird assemblages and medium sized mammals, while among point richness was most important to small mammals (Fig 4a).

Turnover of dominant species among point localities made a larger than expected contribution to evenness for ants, beetles birds, small and large mammals (Fig 4b). Between replicates was only important for spiders, beetles and birds, while land use made a larger than expected contribution for all taxa except for bats and ants. In fact, land use had a negative impact on ant evenness. Similar to ants, beetles and small mammals, dominant bat species differed between villages (Fig 4b). Villages made a positive contribution to evenness of spiders, beetles and small mammals while seasonality did not, suggesting that the same species tend to dominate assemblages across seasons.

## Discussion

Although its impact varied between taxa, land use represented the dominant driver of γ diversity in the region, both for species richness and affecting the distribution of dominant species within taxa. Larger scale processes such as turnover across the region and seasonality were also important to invertebrates, but played a smaller role in vertebrates except maybe for small mammals and birds that displayed a certain amount of seasonality.

### Microhabitat (from points to between replicates)

Most of the bat species or species-groups in the region were observed at all point localities. This is a direct consequence of the larger scale at which these taxa function, and is probably larger than the spatial extent of our study. This very low beta diversity observed for bats could be slightly compounded by the fact that five of the 13 species-groups potentially contained more than one species (due to overlapping echolocation call structure of some species). Surprisingly, this low beta diversity was not true for birds. Turnover among points was particularly important for medium sized mammals, small mammals, ants and spiders and suggests that sampling scales of replicates can be considered as relevant scales during conservation planning for these taxa. Managing local habitat heterogeneity at these scales would therefore further enhance diversity, e.g. importance of local heterogeneity for ants might be linked to the availability of bare ground and canopy cover that varied considerably at this scale [52, 53].

### Land use

Birds and medium sized mammals were the most sensitive to human induced changes. Medium sized mammals in particular drew a very distinct response, almost disappearing from the settlements, increasing in the rangeland and peaking in diversity in the agricultural plots (S1 Fig). Three, not mutually exclusive hypotheses can explain the observed structuring of this. First, rural agricultural areas are normally quite heterogeneous with great seasonal variation in land use [54] which are important drivers in structuring communities due to richer food sources and abundance of breeding and resting space [55]. Secondly, agricultural areas in this and other studies are often dominated by rodent pest species [56], an important food source for avian, reptilian and mammalian species. Thirdly, however, the positive effects of the agricultural matrix depends on structural and ecological integrity of the vegetation [57]. Encroached and overgrazed vegetation as found in the rangelands of this study area, loses its structural integrity which negatively impact on species diversity and abundance [58]. In our system the agricultural areas were the only areas that retained some structural integrity which seems to drive the structuring of mammalian diversity and abundance. As such our results show that anthropologically driven changes in vegetation (e.g. encroached vegetation) can override some predicted ecological changes, e.g. increased small carnivore abundance due to meso-predator release; which can explain some contradicting ecological predictions in agricultural areas [59]. The negative effect of land use on evenness is particularly evident in ants. This response is tied to the dominance of *Anoplolepis custodiens*, a species often linked to disturbance [60], that was detected throughout the landscape, but particularly in the settlements and agricultural plots.

Bats and small mammals were least affected by this scale, although insectivorous bat abundance did increase in the settlements (S1 Fig). This may be due to increased moth, mosquito and other nocturnal insect biomass around lights and people and/or the availability of artificial roosts such as houses and planted trees [49]. At the scale of our study and from the perspective of bat species richness, different bat species seem to perceive the different land use types as a single habitat when commuting and foraging. In contrast to bats, small mammal abundance peaked in the agricultural sites with important implications for associated predator species and cropping systems (S1 Fig). Nevertheless, at least for bats, significant species level and compositional changes were observed due to land use, village and season (PERMANOVA results for 13 species-groups; (S1 File) when considered within the context of foraging group membership (open-air, clutter or clutter-edge; [44] (S1 Table).

Birds, which were the most species rich taxon, also responded positively to the agricultural sites and have been shown to respond positively to increased complexity, structure and vertical organization of habitats [61]. Coetzee and Chown [62] showed that bird diversity responds positively to land use changes but that land use changes precipitate the loss of species that are functionally unique. Interestingly, bird abundance peaked in the settlement and this was mainly the result of large flocks of granivorous birds (e.g. *Lonchura cucullata*). Spiders were the invertebrate taxon that experienced the largest turnover in dominant species between land use types. Croplands were dominated by a nocturnal gnaphosid (*Ibala arcus*), replaced by a diurnal lycosid (*Ocyale guttata*) in the settlements and a stenotopic zodariid species (*Microdiores* sp.) in the rangelands with important implications for spider functional diversity [63]. Spiders are the dominant terrestrial predators [64] and taxa at higher trophic levels are often the most sensitive to disturbance [65]. Beetles almost disappeared from the settlements which could be attributed to the lack of retreats such as logs, dead leaves and litter in these habitats. These features are often burnt or swept up in the villages.

### Regional scale

Relative to ants, regional turnover (among villages) made a smaller, although significant, contribution to spider diversity. Many spider taxa are particularly good dispersers and the distance between these villages did not affect spiders to a similar extent as ants [53]. Except for medium sized mammals, animal assemblages were surprisingly diverse. Diversity in this landscape compared well with that recorded in a range of untransformed habitats (grassland, forest, woodland) in a nearby biodiversity hotspot [66–68]. Such elevated biodiversity may be a function of the landscape heterogeneity created by the combination of human and natural regimes [69]. Alternatively, we could be observing the area’s extinction debt, given the fact that habitat fragmentation in these areas is quite recent [70]. It was also at this scale where the bulk of beetle diversity was generated followed by land use and a strong seasonal component.

The importance of regional diversity for all three invertebrate taxa, points to the importance of historical and dispersal processes in shaping these assemblages and suggests that they would probably be the most affected by an extinction debt. One such an example is that of the discovery of ants in the genus *Prionopelta* inside one of the villages. Populations of this species are locally rare and there are only three species known from tropical Africa [71].

### Seasonality (Time)

The most important driver for both small mammal and beetle assemblages was seasonal turnover. In small mammals this observed pattern is probably linked to seasonal changes in cropping systems and vegetation where rodent abundance and diversity often peak following the rainy season [72] which is probably linked to seasonal changes in crop systems and the trophic link between small mammals and beetles. The major role of seasonality on beetle diversity and more specifically its negative impact on Shannon diversity of this group can be ascribed to the presence of the genus *Zophosis* which is commonly encountered on bare patches of ground in cleared areas of the croplands. Ground beetle presence depends largely on the seasonal availability of food resources; this is especially true for Tenebrionidae and granivorous Harpaline and Chlaeniine carabid taxa, that dominated the beetle assemblages of this study. Furthermore the partitioning of food resources may act to refine the niche space of these beetles [73]. Site-specific mortality would also play an important, albeit unexplored role in the observed distribution of beetles in these landscapes.

Although bats failed to respond to any of the scales investigated, an analysis of bat functional groups suggest that clutter feeders, in particular, had low species richness (only two species) and low abundances throughout all hierarchical levels which could be linked to the loss of vertical structure of vegetation associated with the loss of large trees [74] or absence (or regular disturbance) of roosts (as obligate cave/mine dwellers), e.g. [75]. Similar to ants then, bats seem to respond to processes that are pervasive across the scales that we investigated.

In conclusion, taxon responses in this landscape varied from the loss of functional groups (clutter feeding bats) to dominance of one species (ants) across the landscape, to the decrease (medium sized mammals) and disappearance of whole functional groups in ant [76] and spider assemblages [63] as well as whole taxonomic groups (beetles) in certain land uses. Results from this points to the scales at which animal diversity is affected. Identifying the habitat features (or land use) or keystone structures [3] at these scales that are responsible for generating this diversity would be key to conserving animal biota in the region. It is evident that although the current land use regime in the region that has been around for a short period of time (< 40 years), its footprint influences a considerable amount of the diversity in the region, comparable to processes that operate at larger scales such as regional and temporal turnover.

## Supporting information

S1 Table List of bat species, families and foraging groups recorded from manual identifications of a random subset of four sites (two nights each) per village, and the codes given to species-groups defined for subsequent automated identification with minimal overlap in call parameters using scans and filters in Analook v. 4.1t, 2015 (Titley Electronics, www.hoarybat.com). Single asterisk denotes species which were identified very rarely using manual identification but not detected from automated scans. Double asterisk denotes one species which was not manually detected in the sub-sampled sites but detected unequivocally with the automated scans.
(DOCX)

S2 Table Proportion of total richness contributed by alpha and beta components for all seven taxa based on individual- and sample-based partitioning respectively. (DOCX)

S1 Fig Response of animal communities to three land use types: Croplands, settlements, and rangelands in a rural landscape using two response variables, (a) abundance and (b) richness. All values were standardized for comparison to represent standard deviations from the mean. Whiskers represent the range, boxes the first and third quartiles, dark lines the median and isolated circles are outliers.
(DOCX)

S1 File R-script and associated R output of PERMANOVA analyses for acoustically-obtained (SM2 bat detectors, Wildlife Acoustics) abundance data for 13 species-groups of bats using Bray-Curtis distance. Analyses were conducted in R using the “vegan”, “car” and “MASS” packages. Species group codes and foraging associations (open-air, clutter and clutter-edge; Schoeman & Jacobs, 2008)) are explained in S1 Table.
(DOCX)
