# Supplementary material for: Animal taxa contrast in their scale-dependent responses to land use change in rural Africa
Source: PLoS One. 2018 May 8;13(5):e0194336. doi: 10.1371/journal.pone.0194336 (PMC5940192; doi:10.1371/journal.pone.0194336)
Supplement: S1 Table — Single asterisk denotes species which were identified very rarely using manual identification but not detected from automated scans. Double asterisk denotes one species which was not manually detected in the sub-sampled sites but detected unequivocally with the automated scans. (DOCX) [file pone.0194336.s001.docx]

*PLoS One*

**SUPPLEMENTARY MATERIAL**

Animal taxa contrast in their scale-dependent responses to land use in a modern African cultural landscape

**S1 Table. List of bat species, families and foraging groups recorded from manual identifications of a random subset of four sites (two nights each) per village, and the codes given to species-groups defined for subsequent automated identification with minimal overlap in call parameters using scans and filters in Analook v. 4.1t, 2015 (Titley Electronics, www.hoarybat.com). Single asterisk denotes species which were identified very rarely using manual identification but not detected from automated scans. Double asterisk denotes one species which was not manually detected in the sub-sampled sites but detected unequivocally with the automated scans.**

| Species | Family | Foraging group | Species group code |
| --- | --- | --- | --- |
| *Taphozous mauritianus* | Emballanuridae | Open-air | CP.Ta.Mc.Tm |
| *Hipposideros caffer*** | Hipposideridae | Clutter | Hcaf |
| *Miniopterus natalensis* | Miniopteridae | Clutter-edge | Mn.Pr |
| *Chaerephon ansorgei* | Molossidae | Open-air | Cansor |
| *Chaerephon pumilus* | Molossidae | Open-air | CP.Ta.Mc.Tm |
| *Mops condylurus* | Molossidae | Open-air | CP.Ta.Mc.Tm |
| *Mops midas* | Molossidae | Open-air | Mmidas |
| *Otomops martiensseni* | Molossidae | Open-air | Otomops |
| *Tadarida aegyptiaca* | Molossidae | Open-air | CP.Ta.Mc.Tm |
| *Rhinolopus simulator** | Rhinolophidae | Clutter | Rsim |
| *Eptesicus hottentotus* | Vespertilionidae | Clutter-edge | Ehott |
| *Kerivoula sp* | Vespertilionidae | Clutter | Keriv |
| *Laephotis botswanae* | Vespertilionidae | Clutter-edge | Sd.Lb |
| *Myotis tricolor** | Vespertilionidae | Clutter-edge | Mt* |
| *Myotis welwitschi* | Vespertilionidae | Clutter-edge | Mwel |
| *Neoromicia capensis* | Vespertilionidae | Clutter-edge | Ncap |
| *Neoromicia nanus* | Vespertilionidae | Clutter-edge | Nnanus |
| *Neoromicia zuluensis* | Vespertilionidae | Clutter-edge | Ph.Nz |
| *Pipistrellus hesperidus* | Vespertilionidae | Clutter-edge | Ph.Nz |
| *Pipistrellus rusticus* | Vespertilionidae | Clutter-edge | Mn.Pr |
| *Scotophilus dingani* | Vespertilionidae | Clutter-edge | Sd.Lb |
