# Supplementary material for: Animal taxa contrast in their scale-dependent responses to land use change in rural Africa
Source: PLoS One. 2018 May 8;13(5):e0194336. doi: 10.1371/journal.pone.0194336 (PMC5940192; doi:10.1371/journal.pone.0194336)
Supplement: S2 Table — (DOCX) [file pone.0194336.s002.docx]

*PLoS One*

**SUPPLEMENTARY MATERIAL**

Animal taxa contrast in their scale-dependent responses to land use in a modern African cultural landscape

**S2 Table - Table Proportion of total richness contributed by alpha and beta components for all seven taxa based on individual- and sample-based partitioning respectively.**

Table. Results of individual-based partitioning of species richness and Shannon diversity between four spatial scales and one temporal scale.

| Individual-based |  | Richness | | | Shannon | | |
| --- | --- | --- | --- | --- | --- | --- | --- |
| Taxon | Levels | + | ns | - | + | ns | - |
| Ants | alfa (point) |  |  | 5.4 | 80.6 |  |  |
|  | among point |  |  | 21.8 | 48.5 |  |  |
|  | among replicates |  |  | 10.2 |  | 1.2 |  |
|  | among land uses | 26.5 |  |  |  |  | -27.9 |
|  | among villages | 25.4 |  |  | 2.3 |  |  |
|  | among seasons |  | 10.7 |  |  |  | -4.8 |
| Spiders | alfa (point) |  |  | 2.0 |  |  | 19.4 |
|  | among point |  |  | 16.7 |  |  | 52.7 |
|  | among replicates |  |  | 8.8 | 7.2 |  |  |
|  | among land uses |  | 25.5 |  | 13.1 |  |  |
|  | among villages | 21.6 |  |  | 4.2 |  |  |
|  | among seasons | 25.4 |  |  | 3.4 |  |  |
| Beetles | alfa (point) |  |  | 3.2 |  |  | 27.7 |
|  | among point |  |  | 7.8 | 74.2 |  |  |
|  | among replicates |  | 5.5 |  | 24.1 |  |  |
|  | among land uses |  |  | 22.7 |  | 14.2 |  |
|  | among villages |  | 21.6 |  | 11.2 |  |  |
|  | among seasons | 39.2 |  |  |  |  | -51.3 |
| Bats | alfa (point) |  |  | 93.7 |  |  | 90.5 |
|  | among point | 5.6 |  |  | 4.1 |  |  |
|  | among land uses |  | 0.6 |  | 3.4 |  |  |
|  | among villages |  | 0.0 |  |  | 0.1 |  |
|  | among seasons |  | 0.0 |  | 2.0 |  |  |
| Small mammals | alfa (point) |  |  | 10.4 |  |  | 8.5 |
|  | among point |  |  | 23.3 |  |  | 42.3 |
|  | among replicates |  | 6.6 |  |  | 2.3 |  |
|  | among land uses |  | 22.2 |  | 20.6 |  |  |
|  | among villages | 16.7 |  |  | 17.6 |  |  |
|  | among seasons |  | 20.8 |  | 8.6 |  |  |
| Birds | alfa (point) |  |  | 17.8 |  |  | 74.4 |
|  | among point |  |  | 12.8 | 10.0 |  |  |
|  | among replicates |  |  | 16.3 | 5.9 |  |  |
|  | among land uses | 32.6 |  |  | 6.2 |  |  |
|  | among seasons | 20.5 |  |  | 3.6 |  |  |
| Large mammals | alfa (point) |  |  | 12.6 |  |  | 27.6 |
|  | among point |  |  | 37.4 |  | 49.5 |  |
|  | among land uses | 35.0 |  |  | 18.3 |  |  |
|  | among villages | 15.0 |  |  | 4.6 |  |  |

| Sample-based |  | Richness | | Shannon | |
| --- | --- | --- | --- | --- | --- |
| Taxon | levels | + | ns | + | ns |
| Ants | alfa (point) |  | 5.4 |  | 80.6 |
|  | among points | 21.8 |  | 48.5 |  |
|  | among replicates | 10.2 |  |  | 1.2 |
|  | among land uses | 26.5 |  |  | -27.9 |
|  | among villages | 25.4 |  | 2.3 |  |
|  | among seasons |  | 10.7 |  | -4.8 |
| Spiders | alfa (point) |  | 2.0 |  | 19.4 |
|  | among points | 16.7 |  | 52.7 |  |
|  | among replicates | 8.8 |  | 7.2 |  |
|  | among land uses | 25.5 |  | 13.1 |  |
|  | among villages | 21.6 |  | 4.2 |  |
|  | among seasons |  | 25.4 |  | 3.4 |
| Beetles | alfa (point) |  | 10.6 |  | 82.8 |
|  | among points | 0.3 |  | 19.7 |  |
|  | among replicates | 5.6 |  | 24.0 |  |
|  | among land uses | 22.4 |  | 14.3 |  |
|  | among villages | 21.6 |  | 11.3 |  |
|  | among seasons |  | 39.4 |  | -51.9 |
| Bats | alfa (point) |  | 93.7 |  | 90.5 |
|  | among points | 5.6 |  | 4.5 |  |
|  | among land uses |  | 0.6 |  | 1.8 |
|  | among villages |  | 0.0 |  | 2.5 |
|  | among seasons |  | 0.0 |  | 0.7 |
| Rodents | alfa (point) |  | 10.4 |  | 8.5 |
|  | among points | 23.3 |  | 42.3 |  |
|  | among replicates | 6.6 |  | 2.3 |  |
|  | among land uses | 22.2 |  | 20.6 |  |
|  | among villages | 16.7 |  | 17.6 |  |
|  | among seasons |  | 20.8 |  | 8.6 |
| Birds | alfa (point) |  | 17.6 |  | 74.4 |
|  | among points | 12.8 |  | 10.0 |  |
|  | among replicates | 16.3 |  | 5.9 |  |
|  | among land uses | 32.6 |  | 6.2 |  |
|  | among seasons |  | 20.7 |  | 3.6 |
| Mammals | alfa (point) |  | 7.5 |  | 15.7 |
|  | among points | 32.4 |  | 46.0 |  |
|  | among land uses | 47.0 |  | 33.7 |  |
|  | among villages |  | 13.2 |  | 4.6 |
