# Supplementary material for: Animal taxa contrast in their scale-dependent responses to land use change in rural Africa
Source: PLoS One. 2018 May 8;13(5):e0194336. doi: 10.1371/journal.pone.0194336 (PMC5940192; doi:10.1371/journal.pone.0194336)

*PLoS One*

**SUPPLEMENTARY MATERIAL**

Animal taxa contrast in their scale-dependent responses to land use in a modern African cultural landscape

**S1 Fig. Response of animal communities to three land use types: Croplands, Settlements, and Rangelands in a rural landscape using two response variables, (a) abundance and (b) richness. All values were standardized for comparison to represent standard deviations from the mean. Whiskers represent the range, boxes the first and third quartiles, dark lines the median and isolated circles are outliers.**

S1 Figure


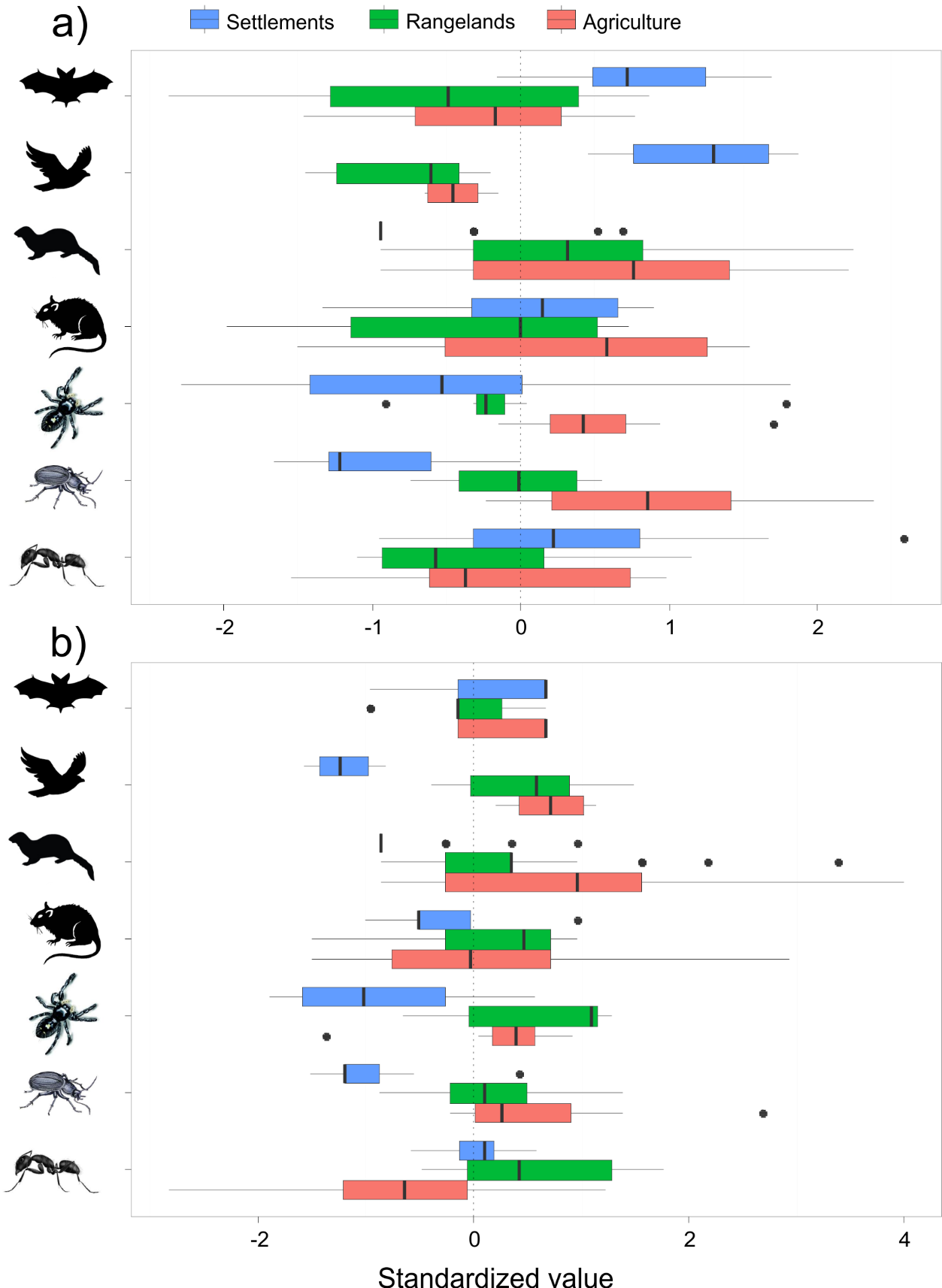

Supplement: S1 Fig — All values were standardized for comparison to represent standard deviations from the mean. Whiskers represent the range, boxes the first and third quartiles, dark lines the median and isolated circles are outliers. (DOCX) [file pone.0194336.s003.docx]
