# Supplementary material for: Animal taxa contrast in their scale-dependent responses to land use change in rural Africa
Source: PLoS One. 2018 May 8;13(5):e0194336. doi: 10.1371/journal.pone.0194336 (PMC5940192; doi:10.1371/journal.pone.0194336)
Supplement: S1 File — Analyses were conducted in R using the “vegan”, “car” and “MASS” packages. Species group codes and foraging associations (open-air, clutter and clutter-edge; Schoeman & Jacobs, 2008)) are explained in S1 Table. (DOCX) [file pone.0194336.s004.docx]

*PLoS One*

**SUPPLEMENTARY MATERIAL**

Animal taxa contrast in their scale-dependent responses to land use in a modern African cultural landscape

**S1 File. R-script and associated R output of PERMANOVA analyses for acoustically-obtained (SM2 bat detectors, Wildlife Acoustics) abundance data for 13 species-groups of bats using Bray-Curtis distance. Analyses were conducted in R using the “vegan”, “car” and “MASS” packages. Species group codes and foraging associations (open-air, clutter and clutter-edge; Schoeman & Jacobs, 2008)) are explained in S1 Table. Data file available on request from PJT**.

MULTIPLE-SPECIES PERMANOVA FOR 13 SPECIES GROUPS BASED ON BRAY-CURTIS DISTANCES

setwd("C:/Users/Prof-Taylor/Dropbox/Papers/LLL hierarchical diversity/Permanova")

require(vegan)

library(car)

require(MASS)

batcounts<-read.csv("batcounts.csv")

attach (batcounts)

test<-batcounts[,1]

landuse<-substr(test,4,4)

village<-substr(test,3,3)

season<-c(rep("a",19),rep("b",19))

transformed<-sqrt(batcounts[,-1])

dist1<-vegdist(transformed)

> anova(betadisper(dist1,village))

Analysis of Variance Table

Response: Distances

Df Sum Sq Mean Sq F value Pr(>F)

Groups 1 0.067413 0.067413 9.8737 0.004733 **

Residuals 22 0.150206 0.006828

---

Signif. codes: 0 ‘***’ 0.001 ‘**’ 0.01 ‘*’ 0.05 ‘.’ 0.1 ‘ ’ 1

> adonis(dist1~landuse*village*season)

Call:

adonis(formula = dist1 ~ landuse * village * season)

Permutation: free

Number of permutations: 999

Terms added sequentially (first to last)

Df SumsOfSqs MeanSqs F.Model R2 Pr(>F)

landuse 2 0.9401 0.47003 4.6613 0.21001 0.001 ***

village 1 1.0972 1.09715 10.8806 0.24510 0.001 ***

season 1 0.2447 0.24474 2.4272 0.05468 0.027 *

landuse:village 2 0.5144 0.25719 2.5506 0.11491 0.002 **

landuse:season 2 0.1884 0.09420 0.9342 0.04209 0.504

village:season 1 0.0834 0.08343 0.8274 0.01864 0.612

landuse:village:season 2 0.1981 0.09906 0.9824 0.04426 0.480

Residuals 12 1.2100 0.10084 0.27032

Total 23 4.4763 1.00000

---

Signif. codes: 0 ‘***’ 0.001 ‘**’ 0.01 ‘*’ 0.05 ‘.’ 0.1 ‘ ’ 1

SPECIES-GROUP LINEAR MODELS OF SQUARE-ROOT TRANSFORMED ABUNDANCE

MN.PR (*Miniopterus natalensis*/ *Pipistrellus rusticus*) – Clutter edge foragers


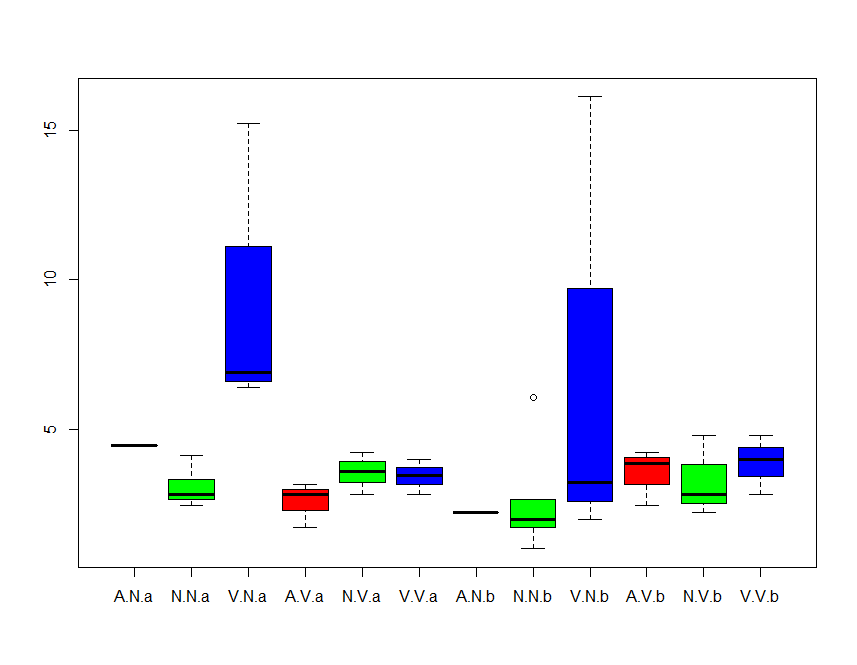


Key:

A.N.a = Agriculture . Ndengeza. Session”a” (Late summer2014)

N.N.a = “Natural”(rangelands). Ndengeza. Session”a” (Late summer2014)

V.N.a = Village. Ndengeza. Session”a”’ (Late summer 2014)

A.V.a= Agriculture . Vyeboom. Session”a” (Late summer2014)

N.V.a= “Natural” (rangelands). Vyeboom. Session”a” (Late summer2014)

V.V.a= Village. Vyeboom. Session”a” (Late summer2014)

A.N.b= Agriculture . Ndengeza. Session”b” (Early summer 2015)

N.N.b= “Natural” (rangelands). Ndengeza. Session”b” (Early summer 2015)

V.N.b= Village. Ndengeza. Session”b” (Early summer 2015)

A.V.b= Agriculture . Vyeboom. Session”b” (Early summer 2015)

N.V.b= “Natural” (rangelands). Vyeboom. Session”b” (Early summer 2015)

V.V.b= Village. Vyeboom. Session”b” (Early summer 2015)

> Anova (Mod_MN.PR)

Anova Table (Type II tests)

Response: Mn.Pr

Sum Sq Df F value Pr(>F)

landuse 64.032 2 3.8576 0.03411 *

village 15.590 1 1.8784 0.18223

season 3.538 1 0.4263 0.51956

landuse:village 36.460 2 2.1965 0.13140

landuse:season 2.474 2 0.1491 0.86226

village:season 7.349 1 0.8855 0.35535

landuse:village:season 4.953 2 0.2984 0.74453

Residuals 215.787 26

---

Signif. codes: 0 ‘***’ 0.001 ‘**’ 0.01 ‘*’ 0.05 ‘.’ 0.1 ‘ ’ 1

PH.NZ (*Pipistrellus hesperidus* / *Neoromica zuluensis*) – Clutter-edge foragers


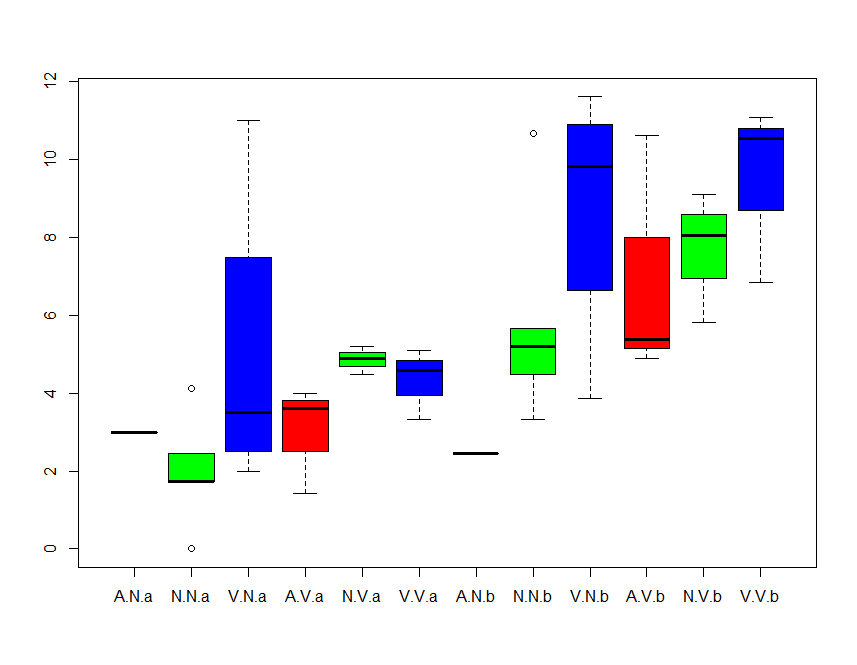


> Anova (Mod_Ph.Nz)

Anova Table (Type II tests)

Response: Ph.Nz

Sum Sq Df F value Pr(>F)

landuse 50.931 2 3.9471 0.0318432 *

village 17.145 1 2.6575 0.1151183

season 127.735 1 19.7989 0.0001437 ***

landuse:village 10.840 2 0.8401 0.4430530

landuse:season 4.111 2 0.3186 0.7299845

village:season 1.659 1 0.2572 0.6163540

landuse:village:season 8.642 2 0.6697 0.5204511

Residuals 167.742 26

---

Signif. codes: 0 ‘***’ 0.001 ‘**’ 0.01 ‘*’ 0.05 ‘.’ 0.1 ‘ ’ 1

SD.LB (*Scotophilus dingani* / *Laephotis botswanae*) Clutter-edge foragers


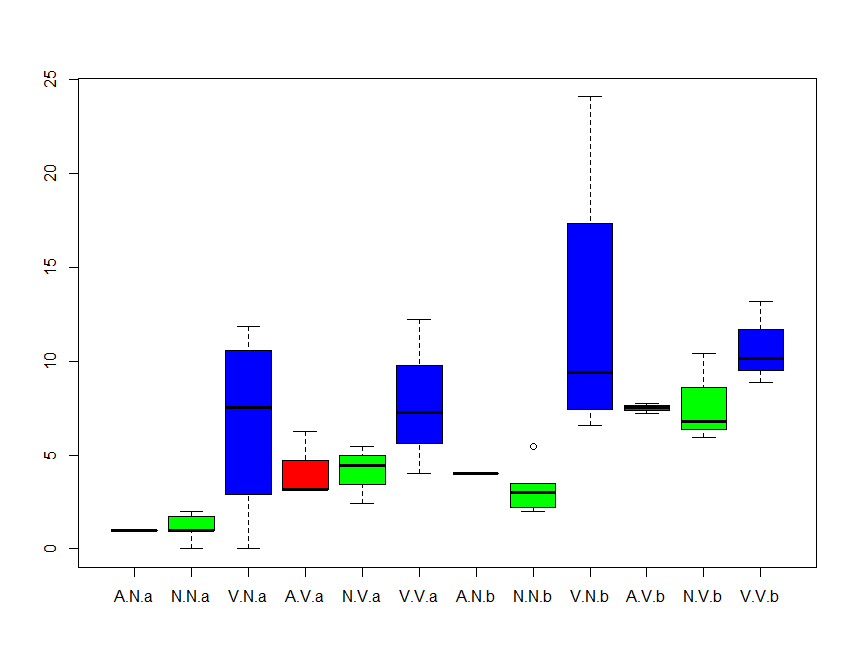


> Anova (Mod_Sd.Lb)

Anova Table (Type II tests)

Response: Sd.Lb

Sum Sq Df F value Pr(>F)

landuse 272.21 2 10.2051 0.0005354 ***

village 37.65 1 2.8226 0.1049238

season 112.14 1 8.4082 0.0074977 **

landuse:village 31.40 2 1.1770 0.3240915

landuse:season 6.31 2 0.2366 0.7909832

village:season 0.32 1 0.0242 0.8776145

landuse:village:season 8.12 2 0.3045 0.7400884

Residuals 346.76 26

---

Signif. codes: 0 ‘***’ 0.001 ‘**’ 0.01 ‘*’ 0.05 ‘.’ 0.1 ‘ ’ 1

CP.TA.MC.TM (*Chaerephon pumilus* / *Tadarida aegyptiaca* / *Mops condylurus* / *Taphozous mauritianus*) Open-air foragers


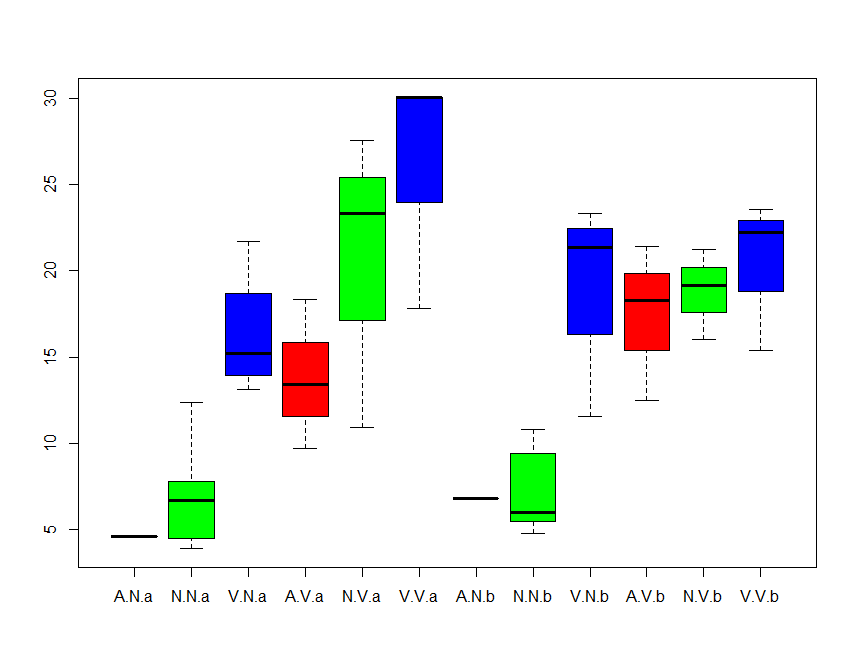


> Anova (Mod_CP.Ta.Mc.Tm)

Anova Table (Type II tests)

Response: CP.Ta.Mc.Tm

Sum Sq Df F value Pr(>F)

landuse 655.72 2 14.6695 5.436e-05 ***

village 739.39 1 33.0825 4.682e-06 ***

season 0.51 1 0.0227 0.8815

landuse:village 93.15 2 2.0838 0.1447

landuse:season 39.63 2 0.8865 0.4242

village:season 35.95 1 1.6086 0.2159

landuse:village:season 33.20 2 0.7426 0.4857

Residuals 581.09 26

---

Signif. codes: 0 ‘***’ 0.001 ‘**’ 0.01 ‘*’ 0.05 ‘.’ 0.1 ‘ ’ 1

Mmidas (*Mops midas*) Open-air foragers


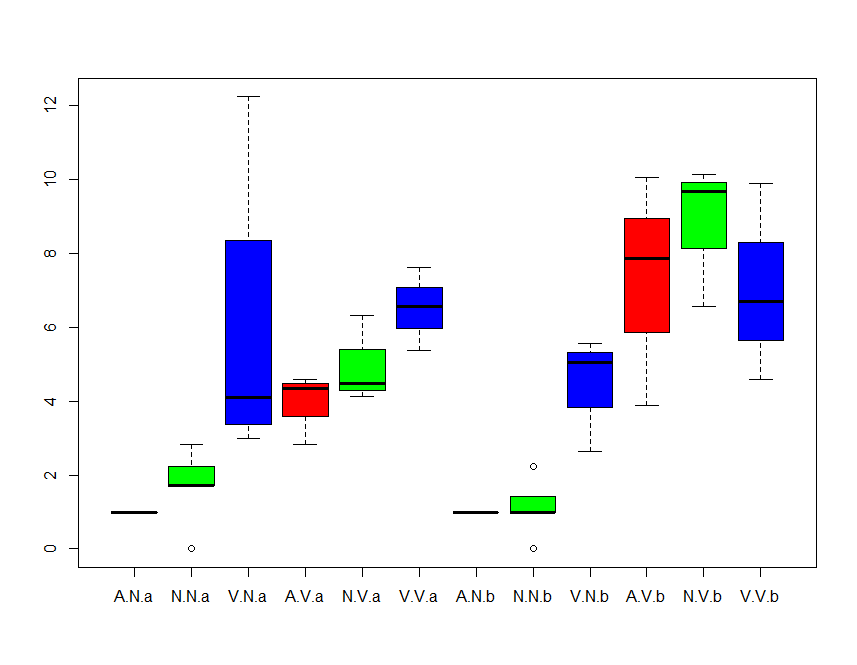


> Anova (Mod_Mmidas)

Anova Table (Type II tests)

Response: Mmidas

Sum Sq Df F value Pr(>F)

landuse 50.091 2 5.5972 0.009516 **

village 123.832 1 27.6739 1.692e-05 ***

season 6.011 1 1.3434 0.256975

landuse:village 28.443 2 3.1782 0.058235 .

landuse:season 7.373 2 0.8239 0.449855

village:season 22.267 1 4.9763 0.034539 *

landuse:village:season 2.970 2 0.3319 0.720553

Residuals 116.342 26

---

Signif. codes: 0 ‘***’ 0.001 ‘**’ 0.01 ‘*’ 0.05 ‘.’ 0.1 ‘ ’ 1

Nnanus (*Neoromicia nana*) Clutter-edge foragers


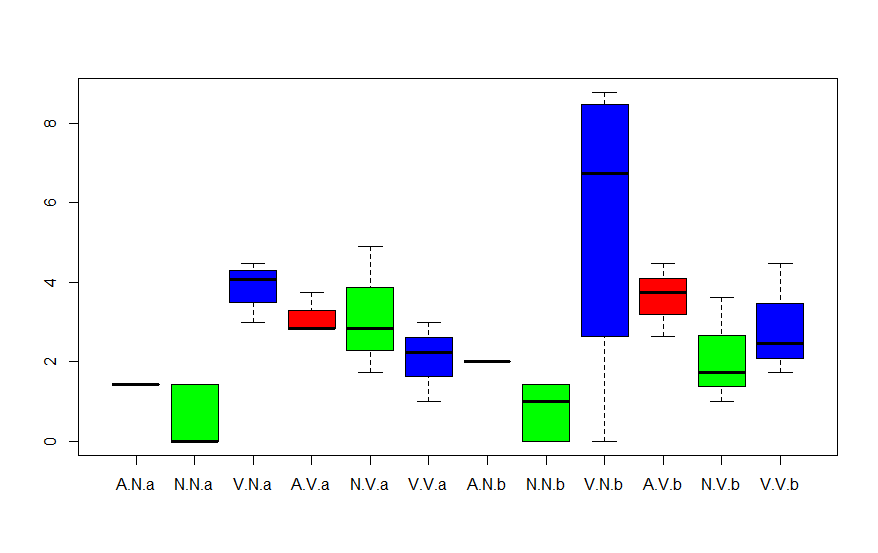


> Anova (Mod_Nnanus)

Anova Table (Type II tests)

Response: Nnanus

Sum Sq Df F value Pr(>F)

landuse 41.760 2 7.6478 0.002443 **

village 0.542 1 0.1985 0.659588

season 2.131 1 0.7806 0.385058

landuse:village 35.495 2 6.5003 0.005137 **

landuse:season 4.985 2 0.9129 0.413825

village:season 1.727 1 0.6326 0.433605

landuse:village:season 0.351 2 0.0643 0.937913

Residuals 70.986 26

---

Signif. codes: 0 ‘***’ 0.001 ‘**’ 0.01 ‘*’ 0.05 ‘.’ 0.1 ‘ ’ 1

Ncap (*Neoromicia capensis*) Clutter-edge foragers


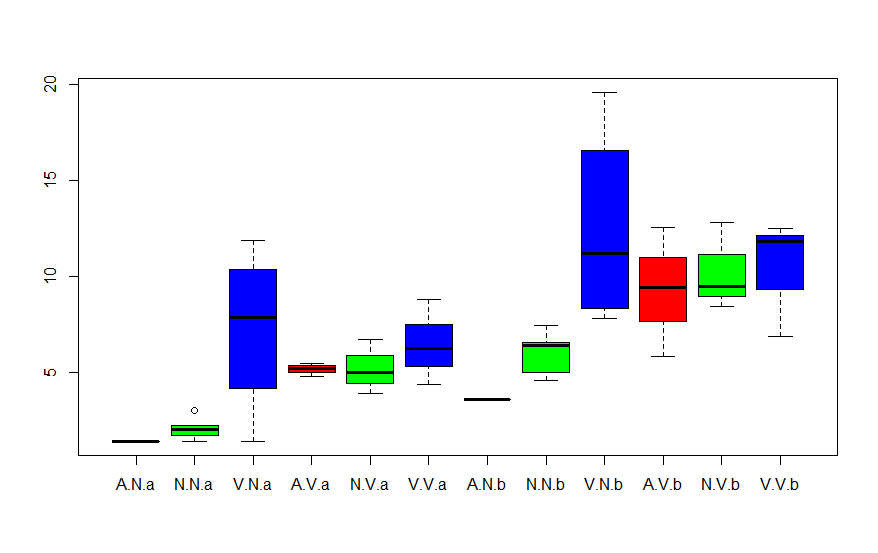


> Anova (Mod_Ncap)

Anova Table (Type II tests)

Response: Ncap

Sum Sq Df F value Pr(>F)

landuse 123.239 2 7.3659 0.002921 **

village 29.405 1 3.5151 0.072089 .

season 176.041 1 21.0437 9.991e-05 ***

landuse:village 61.559 2 3.6793 0.039169 *

landuse:season 1.527 2 0.0912 0.913086

village:season 0.213 1 0.0255 0.874316

landuse:village:season 3.771 2 0.2254 0.799761

Residuals 217.503 26

---

Signif. codes: 0 ‘***’ 0.001 ‘**’ 0.01 ‘*’ 0.05 ‘.’ 0.1 ‘ ’ 1

Ehott (*Eptesicus hottentotus*) Clutter-edge foragers


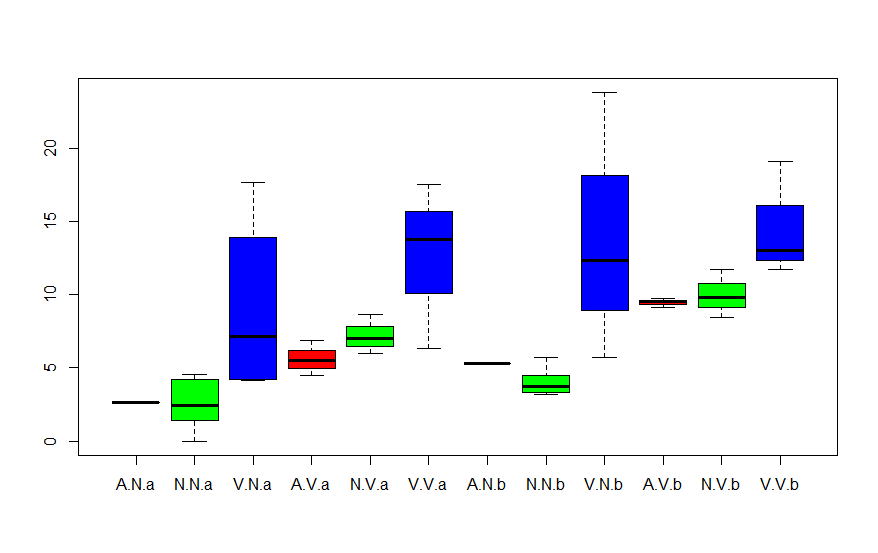


> Anova (Mod_Ehott)

Anova Table (Type II tests)

Response: Ehott

Sum Sq Df F value Pr(>F)

landuse 406.92 2 12.5793 0.0001509 ***

village 126.25 1 7.8057 0.0096479 **

season 78.66 1 4.8634 0.0364770 *

landuse:village 16.31 2 0.5044 0.6096830

landuse:season 5.26 2 0.1626 0.8508190

village:season 0.10 1 0.0063 0.9375032

landuse:village:season 7.02 2 0.2170 0.8063974

Residuals 420.53 26

---

Signif. codes: 0 ‘***’ 0.001 ‘**’ 0.01 ‘*’ 0.05 ‘.’ 0.1 ‘ ’ 1

Mwel (*Myotis welwitschii*) Clutter-edge foragers


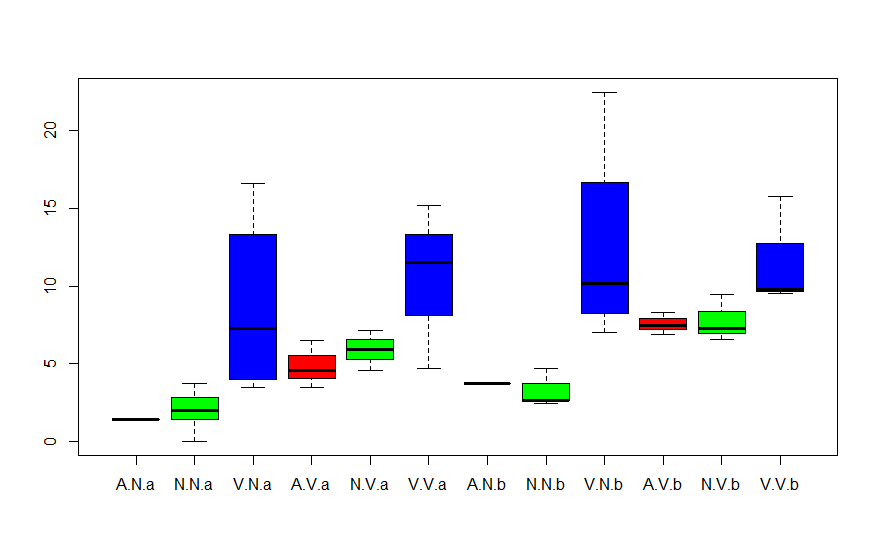


> Anova (Mod_Mwel)

Anova Table (Type II tests)

Response: Mwel

Sum Sq Df F value Pr(>F)

landuse 360.83 2 13.0973 0.0001163 ***

village 61.01 1 4

.4290 0.0451526 *

season 44.57 1 3.2355 0.0836802 .

landuse:village 25.83 2 0.9376 0.4044049

landuse:season 3.82 2 0.1388 0.8710723

village:season 0.99 1 0.0715 0.7912426

landuse:village:season 5.12 2 0.1857 0.8316075

Residuals 358.15 26

---

Signif. codes: 0 ‘***’ 0.001 ‘**’ 0.01 ‘*’ 0.05 ‘.’ 0.1 ‘ ’ 1

Cansor (*Chaerephon cf. ansorgei*) Open-air foragers


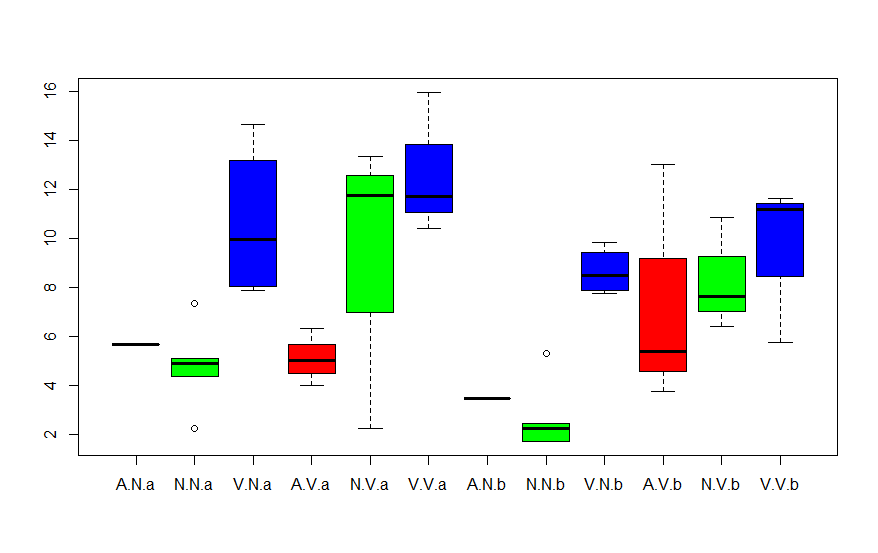


> Anova (Mod_Cansor)

Anova Table (Type II tests)

Response: Cansor

Sum Sq Df F value Pr(>F)

landuse 205.616 2 11.7075 0.0002369 ***

village 78.882 1 8.9829 0.0059263 **

season 17.334 1 1.9740 0.1718603

landuse:village 25.105 2 1.4295 0.2576369

landuse:season 13.609 2 0.7749 0.4711171

village:season 1.571 1 0.1789 0.6757736

landuse:village:season 8.679 2 0.4942 0.6156801

Residuals 228.316 26

---

Signif. codes: 0 ‘***’ 0.001 ‘**’ 0.01 ‘*’ 0.05 ‘.’ 0.1 ‘ ’ 1

Hcaff (*Hipposoderos caffer*) Clutter foragers


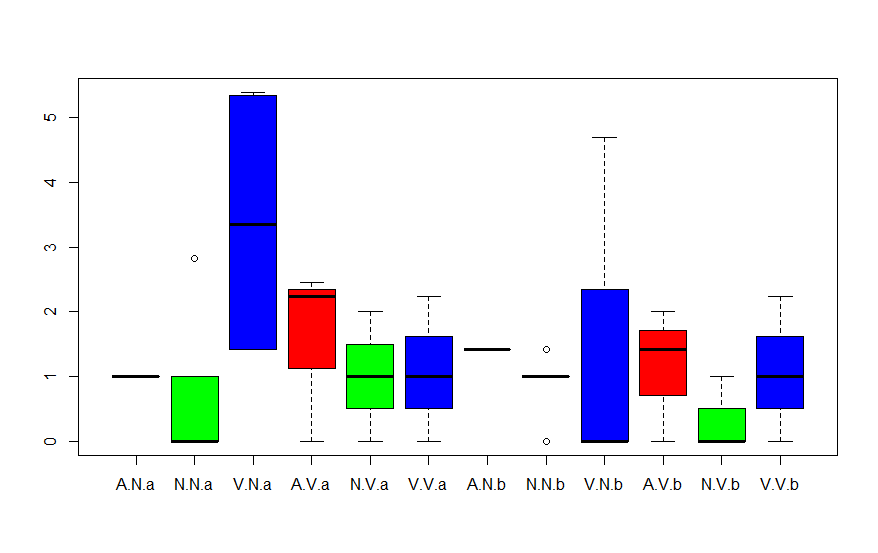


> Anova (Mod_Hcaf)

Anova Table (Type II tests)

Response: Hcaf

Sum Sq Df F value Pr(>F)

landuse 8.173 2 2.0217 0.1527

village 2.309 1 1.1423 0.2950

season 3.234 1 1.6000 0.2171

landuse:village 2.717 2 0.6721 0.5193

landuse:season 2.444 2 0.6045 0.5539

village:season 0.325 1 0.1608 0.6917

landuse:village:season 4.677 2 1.1568 0.3302

Residuals 52.556 26

Keriv (*Kerivoula spp*) Clutter foragers


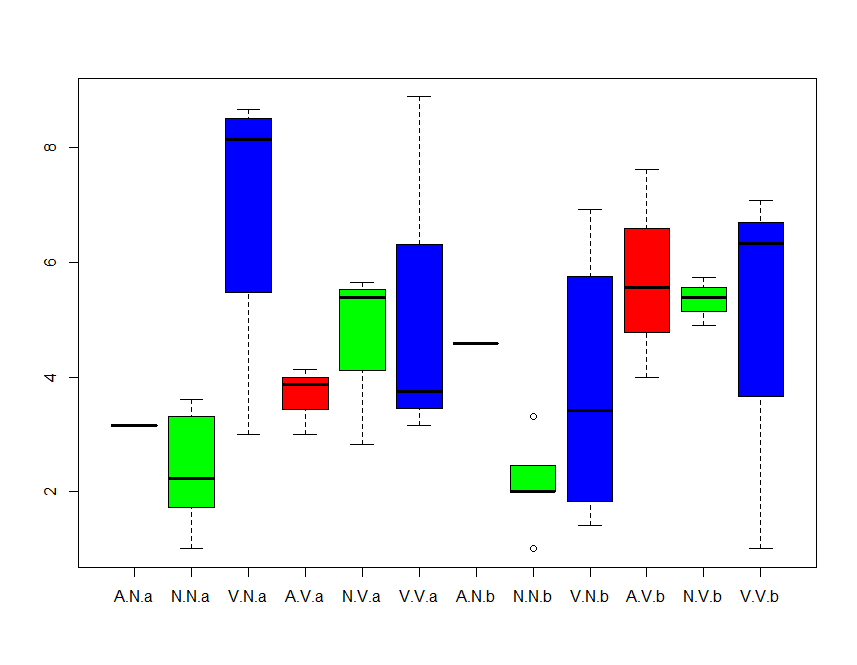


> Anova (Mod_Keriv)

Anova Table (Type II tests)

Response: Keriv

Sum Sq Df F value Pr(>F)

landuse 26.702 2 3.4074 0.04850 *

village 11.976 1 3.0566 0.09221 .

season 0.813 1 0.2075 0.65255

landuse:village 17.179 2 2.1923 0.13188

landuse:season 16.751 2 2.1376 0.13820

village:season 5.552 1 1.4169 0.24468

landuse:village:season 1.852 2 0.2363 0.79121

Residuals 101.873 26

---

Signif. codes: 0 ‘***’ 0.001 ‘**’ 0.01 ‘*’ 0.05 ‘.’ 0.1 ‘ ’ 1

Otomops (*Otomops martiensseni*) Open-air foragers


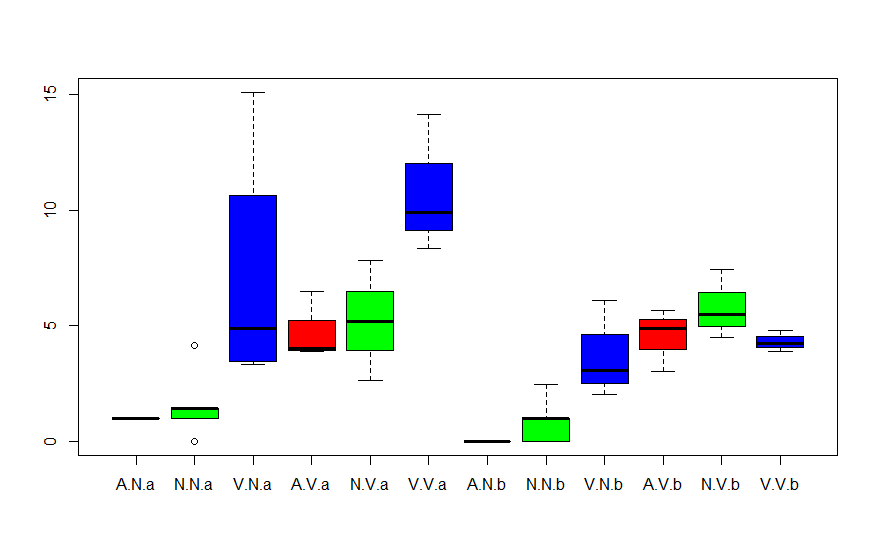


> Anova (Mod_Otomops)

Anova Table (Type II tests)

Response: Otomops

Sum Sq Df F value Pr(>F)

landuse 103.607 2 8.5102 0.0014351 **

village 103.187 1 16.9514 0.0003442 ***

season 36.074 1 5.9262 0.0220900 *

landuse:village 8.158 2 0.6701 0.5202621

landuse:season 44.853 2 3.6842 0.0390207 *

village:season 0.574 1 0.0943 0.7612722

landuse:village:season 8.929 2 0.7334 0.4899549

Residuals 158.268 26

---

Signif. codes: 0 ‘***’ 0.001 ‘**’ 0.01 ‘*’ 0.05 ‘.’ 0.1 ‘ ’ 1
